# Spatial analysis to evaluate risk of malaria in Northern Sumatera, Indonesia

**DOI:** 10.1186/s12936-022-04262-y

**Published:** 2022-08-20

**Authors:** Fahmi Fahmi, Ayodhia Pitaloka Pasaribu, Minerva Theodora, Kinley Wangdi

**Affiliations:** 1 Department of Electrical Engineering, Universitas Sumatera Utara, Medan, 20155 Indonesia; 2 Department of Child Health, Medical Faculty, Universitas Sumatera Utara, Medan, 20155 Indonesia; 3 Directorate of Vector Borne and Zoonotic Disease Control, Ministry of Health of Indonesia, South Jakarta, 12950 Indonesia; 4 Department of Global Health, National Centre for Epidemiology and Population Health, College of Health and Medicine, Australian National University, Acton, ACT 2601 Australia

**Keywords:** Bayesian analysis, Elimination, Indigenous, Indonesia, Imported, Malaria, Northern Sumatera, Spatial analysis

## Abstract

**Background:**

As Indonesia aims for malaria elimination by 2030, provisional malaria epidemiology and risk factors evaluation are important in pursue of this national goal. Therefore, this study aimed to understand the risk factor of malaria in Northern Sumatera.

**Methods:**

Malaria cases from 2019 to 2020 were obtained from the Indonesian Ministry of Health Electronic Database. Climatic variables were provided by the Center for Meteorology and Geophysics Medan branch office. Multivariable logistic regression was undertaken to understand the risk factors of imported malaria. A zero-inflated Poisson multivariable regression model was used to study the climatic drivers of indigenous malaria.

**Results:**

A total of 2208 (indigenous: 76.0% [1679] and imported: 17.8% [392]) were reported during the study period. Risk factors of imported malaria were: ages 19–30 (adjusted odds ratio [AOR] = 3.31; 95% confidence interval [CI] 1.67, 2.56), 31–45 (AOR = 5.69; 95% CI 2.65, 12.20), and > 45 years (AOR = 5.11; 95% CI 2.41, 10.84). Military personnel and forest workers and miners were 1,154 times (AOR = 197.03; 95% CI 145.93, 9,131.56) and 44 times (AOR = 44.16; 95% CI 4.08, 477,93) more likely to be imported cases as compared to those working as employees and traders. Indigenous *Plasmodium falciparum* increased by 12.1% (95% CrI 5.1%, 20.1%) for 1% increase in relative humidity and by 21.0% (95% CrI 9.0%, 36.2%) for 1 °C increase in maximum temperature. *Plasmodium vivax* decreased by 0.8% (95% CrI 0.2%, 1.3%) and 16.7% (95% CrI 13.7%, 19.9%) for one meter and 1 °C increase of altitude and minimum temperature. Indigenous hotspot was reported by Kota Tanjung Balai city and Asahan regency, respectively. Imported malaria hotspots were reported in Batu Bara, Kota Tebing Tinggi, Serdang Bedagai and Simalungun.

**Conclusion:**

Both indigenous and imported malaria is limited to a few regencies and cities in Northern Sumatera. The control measures should focus on these risk factors to achieve elimination in Indonesia.

**Supplementary Information:**

The online version contains supplementary material available at 10.1186/s12936-022-04262-y.

## Background

In 2020, globally there were an estimated 241 million malaria and 627,000 malaria deaths worldwide cases in 87 malaria endemic countries [1]. The World Health Organization (WHO) South-East Asia Region (SEAR) accounted for about 2% of the global malaria burden in the same year [1]. Malaria cases decreased to 724,000 in 2019 from 1,819,000 in 2000 in Indonesia [2]. During the same period malaria deaths decrease to 1170 from 1898 [2]. In line with the *Global Technical Strategy for Malaria 2016–2030* (GTS) [3] and the Roll Back Malaria advocacy plan, *Action and Investment to Defeat Malaria 2016–2030* (AIM) [4], Indonesia has set a national goal of malaria elimination by 2030 [5].

In 2020, 318 out of 576 (62%) districts in Indonesia are classified as malaria-free areas, and 80.4% of Indonesia’s total population are already living in malaria-free zone. However, after 2014 there is a trend of stagnant decrease of malaria cases throughout Indonesia which indicate that malaria control has reached its most difficult stage [6]. The Ministry of Health, Indonesia, Directorate of Disease Control, The Indonesian government has been committed to achieving malaria elimination by 2030 and has developed several strategies such as accelerating the reduction number of malaria cases, intensifying control to eliminate malaria residual foci, preventing the reintroduction in malaria-free areas and environmental management to reduce the influence of climatic on the transmission and vector breeding [7, 8]. In North Sumatera, malaria annual parasite incidence in 2020 was 0.07. Twenty-one out of 33 regencies/cities have been classified as malaria free in 2020 [8]. However, difficult geographical region and high mobility of the people continue to the main source of transmission and challenges malaria elimination efforts in North Sumatera.

In pre-elimination settings, malaria transmission is characterised by clustering of cases in transmission “hotspots” driven by climatic, ecological and human factors [9–11] often in hard-to-reach areas [12]. Therefore, interventions should be focused in areas with higher incidence of malaria than uniform resource allocation for greater effectiveness. In addition, importation of infection should be prevented in areas where malaria has been eliminated [13, 14]. To understand the epidemiological drivers of malaria, stratified analysis of malaria including indigenous, imported and species are important to devise appropriate control and elimination efforts. In addition, identification of malaria hotspots can help delineate problem areas, which can be further investigated to pinpoint possible causes of relatively higher incidences of malaria in a particular area [15]. Hot spots analysis can be done through spatial epidemiological tools (including Geographical Information Systems [GIS] and spatial analytic methods) [16–18].

Therefore, this study aimed to identify risk factors of imported malaria and quantify climatic drivers of indigenous malaria. Secondary aims included identification of indigenous and imported malaria hot spots at regency level. The information for this study can be used to intensify control measures to achieve malaria elimination goals in Indonesia.

## Methods

### Study site

**Fig. 1:**
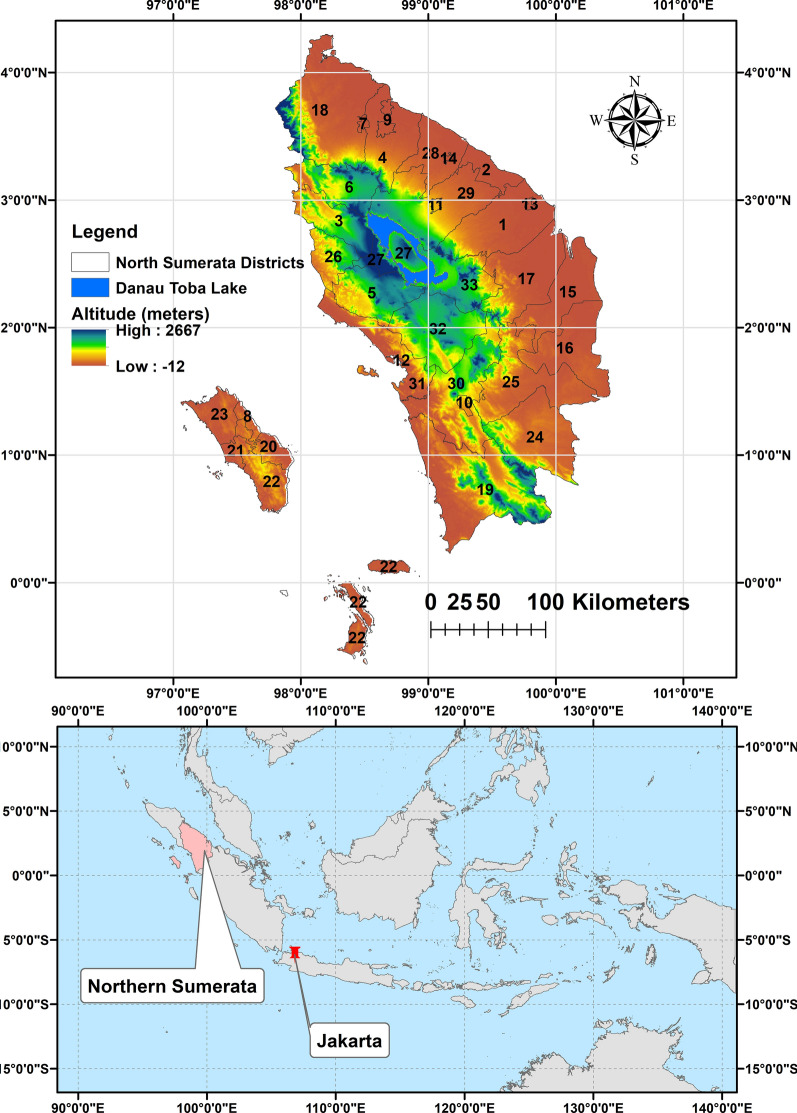
Map of Northern Sumatera, Indonesia with malaria transmitting regencies. 1—Asahan, 2—Batu Bara, 3—Dairi, 4—Deli Serdang, 5—Humbang Hasundutan, 6—Karo, 7—Binjai, 8—Gunungsitoli, 9—Medan, 10—Padangsidimpuan, 11—Pematangsiantar, 12—Sibolga, 13—Tanjungbalai, 14—Tebing Tinggi, 15—Labuhanbatu, 16—Labuhanbatu Selatan, 17—Labuhanbatu Utara, 18—Langkat, 19—Mandailing Natal, 20—Nias, 21—Nias Barat, 22—Nias Selatan, 23—Nias Utara, 24—Padang Lawas, 25—Padang Lawas Utara, 26—Pakpak Bharat, 27—Samosir, 28—Serdang Bedagai, 29—Simalungun, 30—Tapanuli Selatan, 31—Tapanuli Tengah, 32—Tapanuli Utara, 33—Toba

North Sumatera is a province of Indonesia located in the northern part of the Sumatera island. It is located between 0.589° S and 101.0° E and the capital and the largest city is Medan (Fig. 1). North Sumatera is the fourth most populous in Indonesia. The province covers an area of 72,981 km^2^ and a population of 14,799,361 (statistics data 2021). Northern Sumatera is divided into 33 cities and regencies: 1—Asahan, 2—Batu Bara, 3—Dairi, 4—Deli Serdang, 5—Humbang Hasundutan, 6—Karo, 7—Binjai, 8—Gunungsitoli, 9—Medan, 10—Padangsidimpuan, 11—Pematangsiantar, 12—Sibolga, 13—Tanjungbalai, 14—Tebing Tinggi, 15—Labuhanbatu, 16—Labuhanbatu Selatan, 17—Labuhanbatu Utara, 18—Langkat, 19—Mandailing Natal, 20—Nias, 21—Nias Barat, 22—Nias Selatan, 23—Nias Utara, 24—Padang Lawas, 25—Padang Lawas Utara, 26—Pakpak Bharat, 27—Samosir, 28—Serdang Bedagai, 29—Simalungun, 30—Tapanuli Selatan, 31—Tapanuli Tengah, 32—Tapanuli Utara, 33—Toba (Fig. 1).

## Data source

### Malaria

Malaria data of North Sumatera was collected from Indonesian Ministry of Health. The data were extracted from the electronic database from the year 2019–2020. It consists of the name of the regency or city, name of the health centre, demographic data such as age, sex, the month of visit, occupation, address, malaria species, type of malaria examination, outcome and type of transmission (indigenous/imported). Indigenous malaria case (referred to as indigenous case) was defined as those cases without a history of travel outside their place of residence within the last 3 weeks or no contact with imported cases [19]. Imported malaria case (referred to as imported case), the origin of which can be traced to a known malarious area outside the district or municipality in which the case was diagnosed [19]. An electronic map of regency boundaries in shapefile format was obtained from the Global Administrative Areas database (http://www.gadm.org/country).

### Climate

Climate data was obtained from the Center for Meteorology and Geophysics (Badan Meteorologi, Klimatologi and Geofisik—BMKG) Medan branch office. Data from 10 (ten) weather monitoring stations were collected through 2019 and 2020; coming from Sampali Station, Belawan Station, Kualanamu Station, Tuntungan Station, Ngumban Surbakti Station, Sibolga Station, Aek Godang Station, Binaka Station, Onowembo Station and Silangit Station. The collected data was then distributed to obtain climate data in 33 regencies and cities in North Sumatera. The climate data included monthly rainfall, relative humidity level, maximum and minimum temperatures. The altitude of municipalities was obtained from the DIVA-GIS database (https://www.diva-gis.org/). Municipality polygon was used to extract the mean altitude using Zonal statistics in ArcMap 10.7.1 (ESRI Inc., Redlands, CA, USA).

### Population

Population data is obtained from the Central Statistics Agency (Badan Pusat Statistik—BPS) of North Sumatera. The population data were stratified by city and regency for 2019 and 2020 in North Sumatera province. The population was further stratified into male and female population data. A total of 25 regencies and 8 cities were included in this study.

### Spatial autocorrelation analysis

At a global (study area) scale, Moran’s I statistic was used to explore spatial autocorrelation and its strength and to test the assumption of spatial independence. The Getis-Ord Gi* statistic was used to undertake local (regency) level clustering and hot spots analysis [20]. The local Gi* statistic indicates the intensity and stability of hot spot/cold spot clusters [21, 22]. The G* statistic compares the local mean rate (i.e., the rates of a target regency and its neighbourhood regencies) to the global mean rate (the rates for all regencies). The Gi* statistics contain a Z-score and *p*-value of each regency to indicate whether the local and global means are significantly different or not. Regency with a statistically significant and larger Z-score will have a more intense cluster of high values (hot spot), where it is very unlikely that the spatial clustering of high values is the result of a random spatial process; and regencies with a statistically significant and smaller Z-score will have more intense clustering of low values (cold spots) [22].

### Crude standardized morbidity ratios

Crude standardized morbidity ratios (SMRs) analysis of both the species were undertaken to describe the malaria incidence by regency across the study period (2 years). SMR was calculated from:

$${Y}_{i}= \frac{{O}_{i}}{{E}_{i}}$$

*Y*_*i*_ is the overall SMR in regency *i, O*_*i*_—the total number of reported malaria cases in the regency *i* and *E*_*i*_*-* expected number of malaria cases in the regency *i*. The *E*_*i*_ was derived by multiplying the average population for regency *i* with the national incidence of malaria [23].

### Statistical analysis

The risk factor of imported malaria was assessed using logistic regression. The covariates included were sex, age, occupation, malaria species and year of reporting. The number of zero counts for *P. falciparum* and *P. vivax* was 1477/1584 (93.3%) and 1387/1584 (87.6%). Therefore, an analysis to determine the best model. Zero-inflated Poisson (ZIP) regression was selected over the standard Poisson regression because ZIP had a better fit with lower AIC and BIC as compared to Poisson regression, and a Vuong test showed the two models were statically different (Additional file 1: Appendix Tables 1 and 2). Bayesian statistical software WinBUGS version 1.4 (Medical Research Council, Cambridge, UK and Imperial College London, UK) was used to run ZIP regression models for *P. falciparum* and *P. vivax*. The models were tested for each species; the explanatory covariates sex, relative humidity, rainfall and maximum and minimum temperature. The model, which had as an outcome the observed counts of malaria, *Y*, for *i*th regency (*i* = 1…24) in the *j*th month (January 2019-December 2020), and sex *k* was structured as follows:

$$ P(Y_{{ijkl}}  = y_{{ijkl}} ) = \left\{ \begin{gathered}   \omega  + 1\left( {1 - \omega } \right)e^{{ - \mu }} ,y_{{ijkl}}  = 0 \hfill \\   \left( {1 - \omega } \right)e^{{ - \mu }} \mu _{{ijkl}}^{{y_{{ijkl}} }} /y_{{ijkl}} ,y_{{ijkl}} \, > \,0; \hfill \\  \end{gathered}  \right. $$

$$ Y_{ijkl} \sim {\text{Poisson }}\left( {\mu_{ijkl} } \right) $$

$$ {\text{log }}\left( {\mu_{ijkl} } \right) \, = {\text{ log}}\left( {{\text{E}}_{ijkl} } \right) \, + \theta_{ijkl} $$

$$ \theta_{ijkl} = \, \alpha + \beta_{1} \times {\text{ Sex}}_{k} + \beta_{2} \times {\text{ Rh}}_{ij} + \, \beta_{3} \times {\text{ rainfall}}_{ij} + \, \beta_{4} \times {\text{ Tempmax}}_{ij} + \beta_{5} \times {\text{ Tempmin}}_{ij} + {\text{ u}}_{i} $$

where E is the expected number of cases (acting as an offset to control for population size) and θ is the mean log relative risk (RR); α is the intercept, and *β*_*1*_*, β*_*2*_*, β*_*3*_*, β*_*4*_ and *β*_*5*_ are the coefficients for sex (female reference), relative humidity, rainfall and maximum and minimum temperature, respectively; u_*i*_ is the unstructured random effect (assumed to have a mean of zero and variance σ_u_^2^).

A flat prior distribution was specified for the intercept, whereas a normal prior distribution was specified for the coefficients. The priors for the precision of unstructured and spatially structured random effects were specified using non-informative gamma distributions with shape and scale parameters equal to 0.01. Models were also developed with unstructured random effects to assess whether inclusion of these components improved model fit.

An initial burn-in of 10,000 iterations was run and these iterations were discarded. Subsequent blocks of 20,000 iterations were run and examined for convergence. Convergence was assessed by visual inspection of posterior density and history plots and occurred at approximately 100,000 iterations for each model. Ten thousand values from the posterior distributions of each model parameter were stored and summarised for the analysis (posterior mean and 95% CrI).

In all analyses, an α-level of 0.05 was adopted to indicate statistical significance (as indicated by 95% CrI for RR that excluded (1). ArcMap 10.5 software (ESRI, Redlands, CA) was used to generate maps and run hot spot analysis. Stata version 16 (Stata Corporation, College Station, TX, USA) software.

## Results

### Descriptive analysis

A total of 2,208 malaria cases were reported during the study period. There were 54.3% (1198) cases in 2019 and 62.1% (1371) were in males. Seventy-three per cent (1613), 14.3% (216), 12.6% (277) of cases were *P. vivax*, *P. falciparum* and mixed cases (*P. falciparum* and *P. vivax*). There was one case each of *Plasmodium malariae* and *Plasmodium ovale*. Mean age of patients were 24.7 years (range 1–83). Most (26.0%, 574) were in the age group of 19–30 years followed by 13–18 (22.2%, 490) and < 13 (21.5%, 475), respectively. Students (37.4%, 825) and housewives (15.8%, 348) were the two most common occupations. Microscopy diagnoses comprised 54.7% (1208) of cases while rest were diagnosed with the rapid diagnostic test kits.

There were 76.0% (1679) and 17.8% (392) cases of indigenous and imported cases, respectively. Around 6.2% (137) of cases were discarded from the final analysis because the types of cases were not recorded. Males made up 82.6% (324) cases of imported cases with a mean age was 30.5 years (range 1–74 years). Nearly two-thirds of cases were in 19–45 year range followed by 19–30 (45.9%, 180) and 31–45 (31.6%, 124) years. Military personnel made up 48.7% (191) of imported cases. Sixty-four per cent (249) of imported cases were *P. vivax*. Mean altitude, rainfall and relative humidity were 370 m (range 4–1404), 232.5 mm (range 8.4–616.6) and 85.1% (range 75.6 to 95.9), respectively. Mean maximum and minimum temperature during the study was 32.8 °C (range 24.8–37.1) and 20 °C (range 12.7–25.0).

**Fig. 2:**
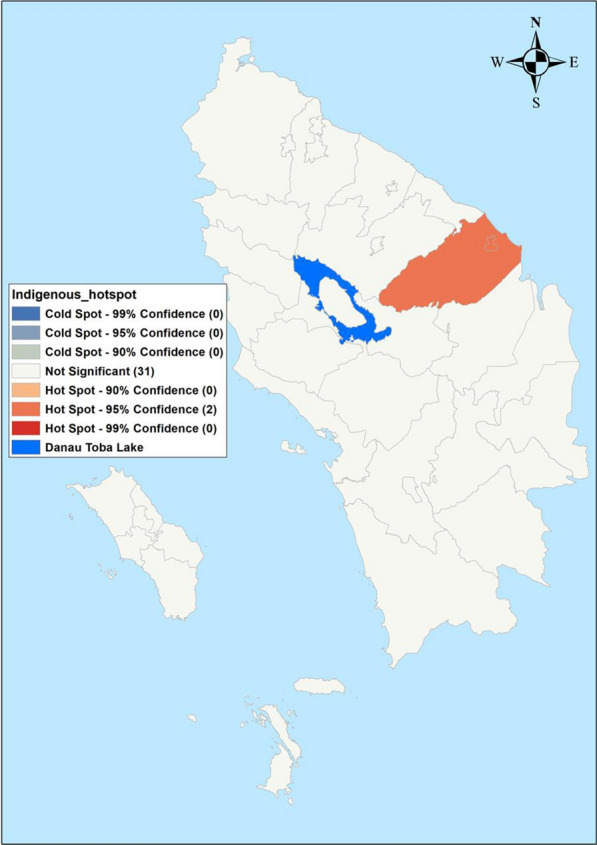
Raw standardized morbidity ratios of **A** *Plasmodium falciparum* and **B** *Plasmodium vivax* by regencies in Northern Sumatera, Indonesia from 2019–2020. 1—Asahan, 2—Batu Bara, 8—Gunungsitoli, 12—Sibolga, 17—Labuhanbatu, 18—Langkat, 19—Mandailing Natal, 20—Nias, 21—Nias Barat, 23—Nias Utara

**Fig. 3:**
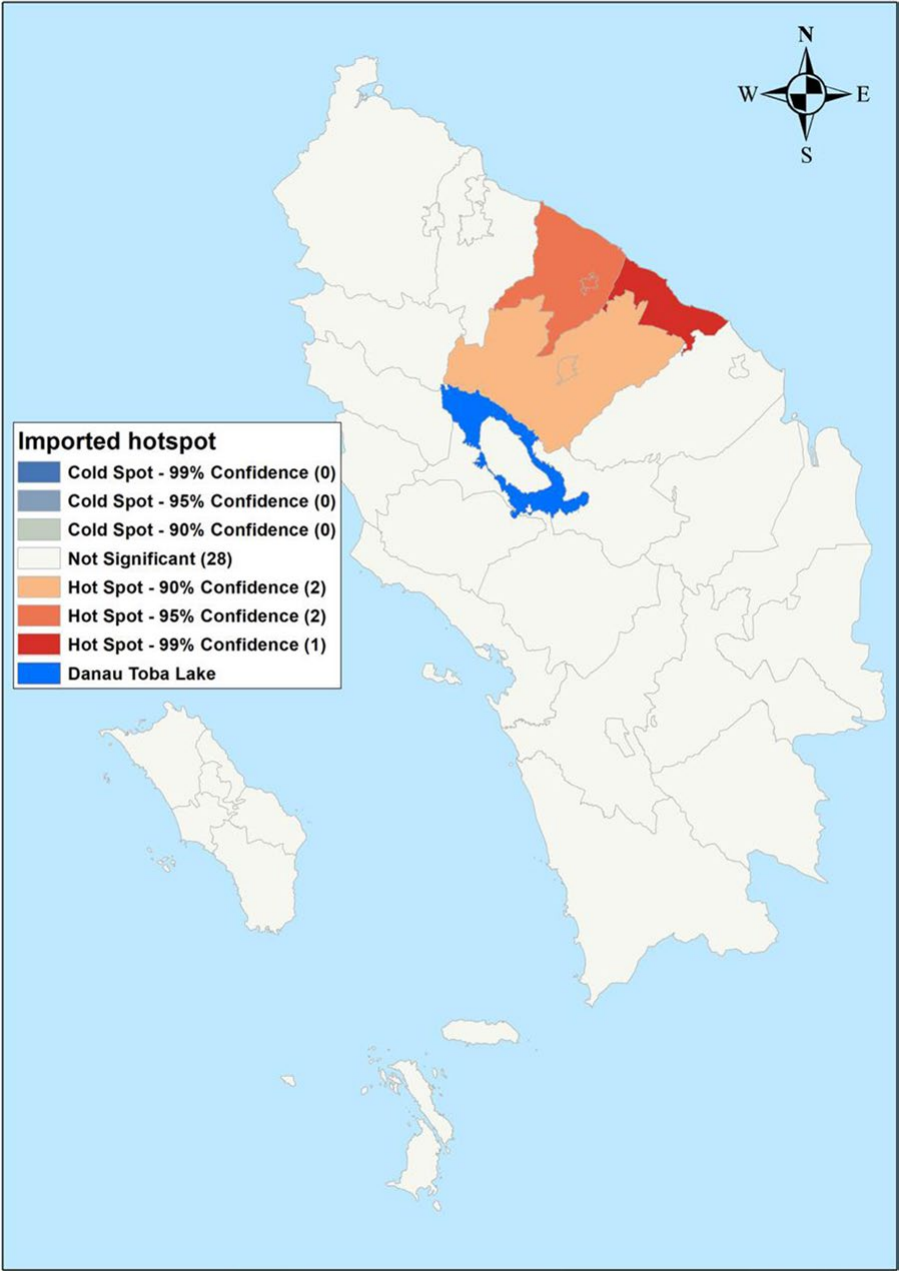
Indigenous cases by regencies in Northern Sumatera, Indonesia from 2019–2020. 1—Asahan, 2—Batu Bara, 8—Gunungsitoli, 12—Sibolga, 17—Labuhanbatu, 18—Langkat, 19—Mandailing Natal, 20—Nias, 21—Nias Barat, 23—Nias Utara

**Fig. 4:**
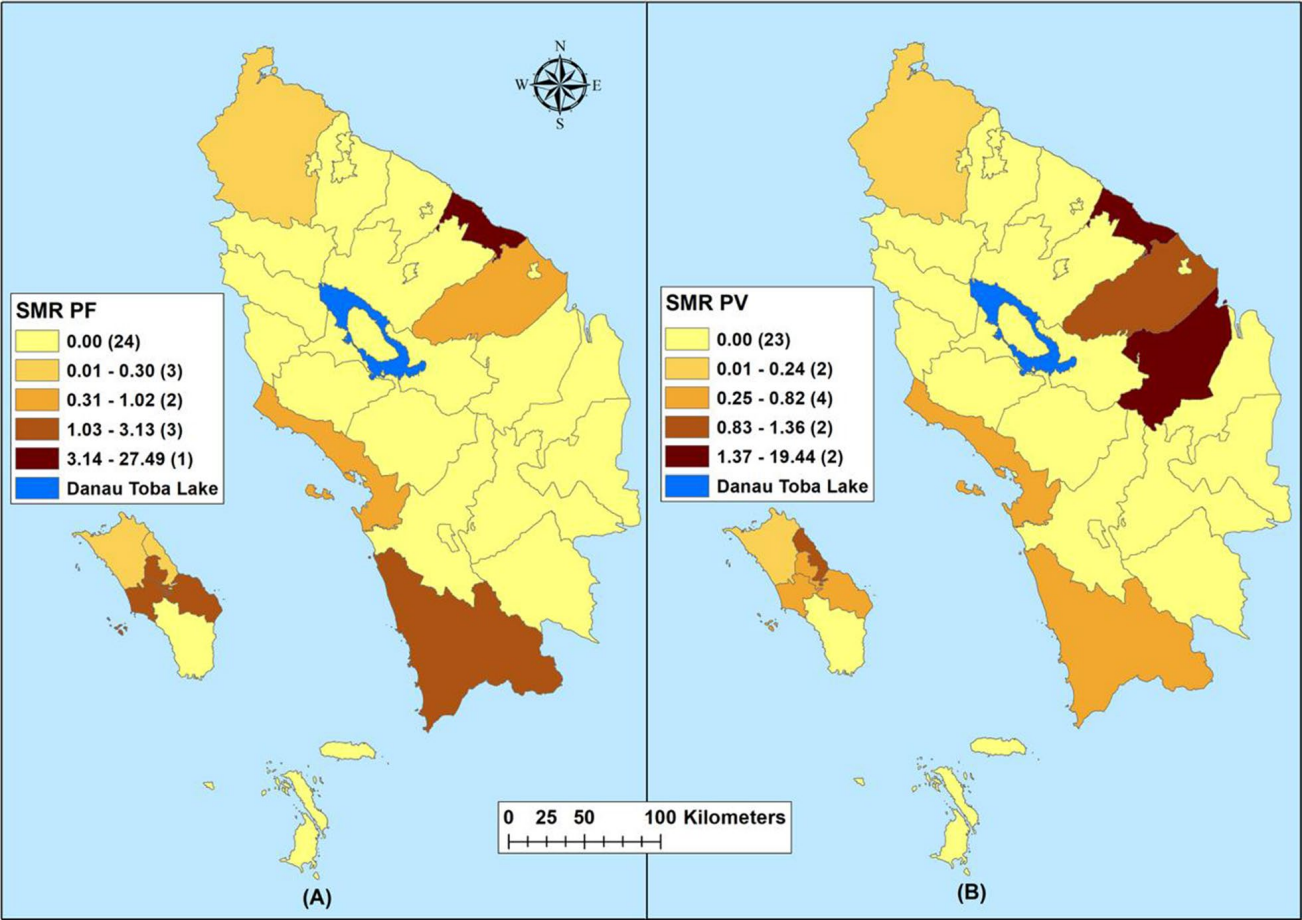
Hot spots (Getis-Ord Gi*) of indigenous cases in Northern Sumatera, Indonesia from 2019–2020. 11—Pematangsiantar, 13—Tanjungbalai

Maps of crude SMRs for *P. falciparum* varied between 0.0 and 27.49 during the study period. Batu Bara Regency reported the highest SMR (31.4–27.49) and 24 regencies reported an SMR of zero cases (Fig. 2). Crude SMR for *P. vivax* varied from 0.0 to 19.44 with Batu Bara and Labuhan Batu Utara regencies reporting the highest SMR (1.37- 19.44). Twenty-three regencies reported an SMR of zero for *P. vivax* (Fig. 2). Indigenous malaria cases hotspot was limited to Asahan and Tanjungbalai regencies (Fig. 3), while imported cases were reported in five adjacent regencies: Batu Bara, Kota Pematang Siantar, Tebing Tinggi, Serdang Bedagai and Simalungun (Fig. 4).

### Hotspot of indigenous and imported malaria

**Fig. 5:**
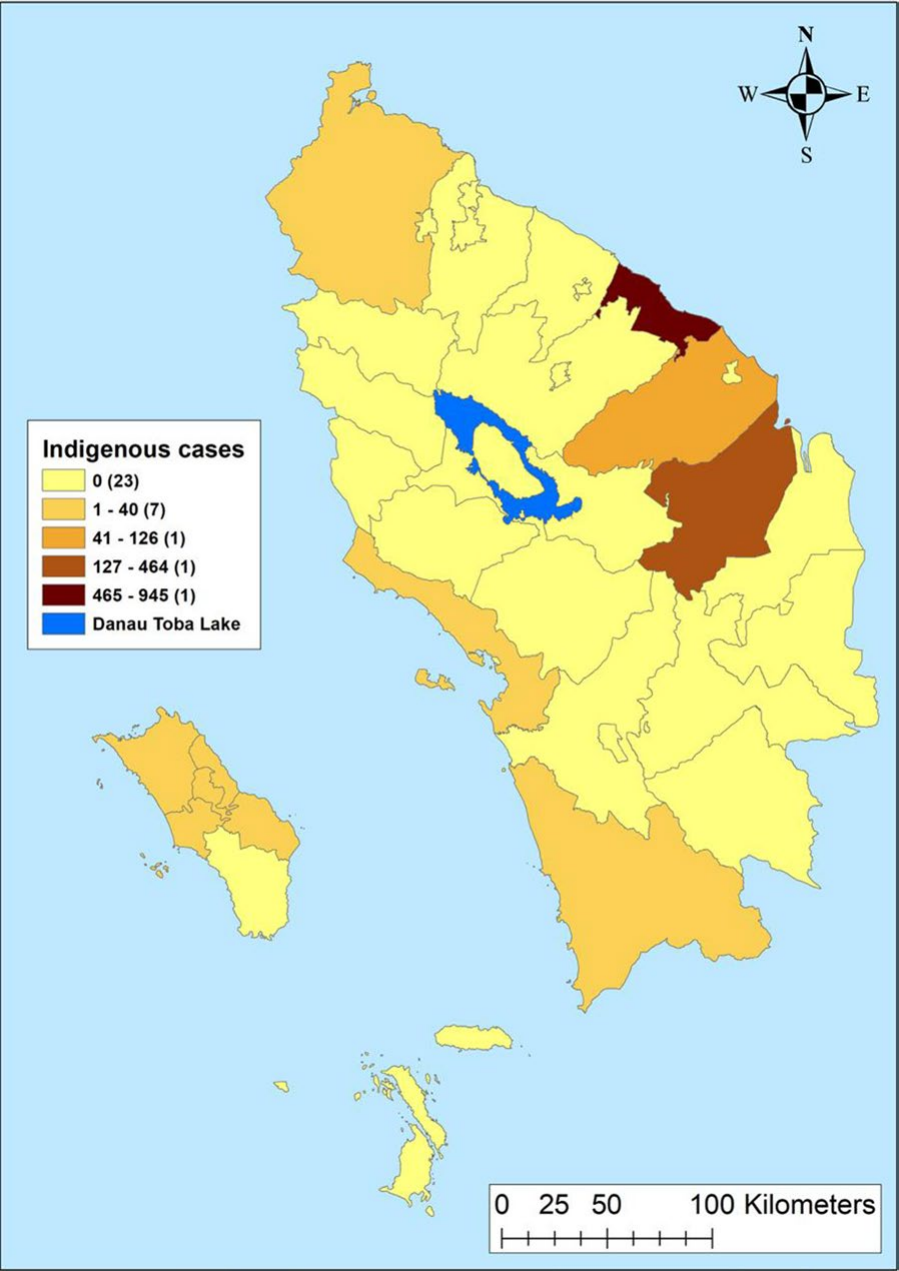
Imported cases by regencies in Northern Sumatera, Indonesia from 2019–2020. 1—Asahan, 2—Batu Bara, 4—Deli Serdang, 7—Binjai, 8—Gunungsitoli, 11—Pematangsiantar, 12—Sibolga, 18—Langkat, 19—Mandailing Natal, 20—Nias, 21—Nias Barat, 23—Nias Utara, 27—Samosir, 33—Toba

**Fig. 6:**
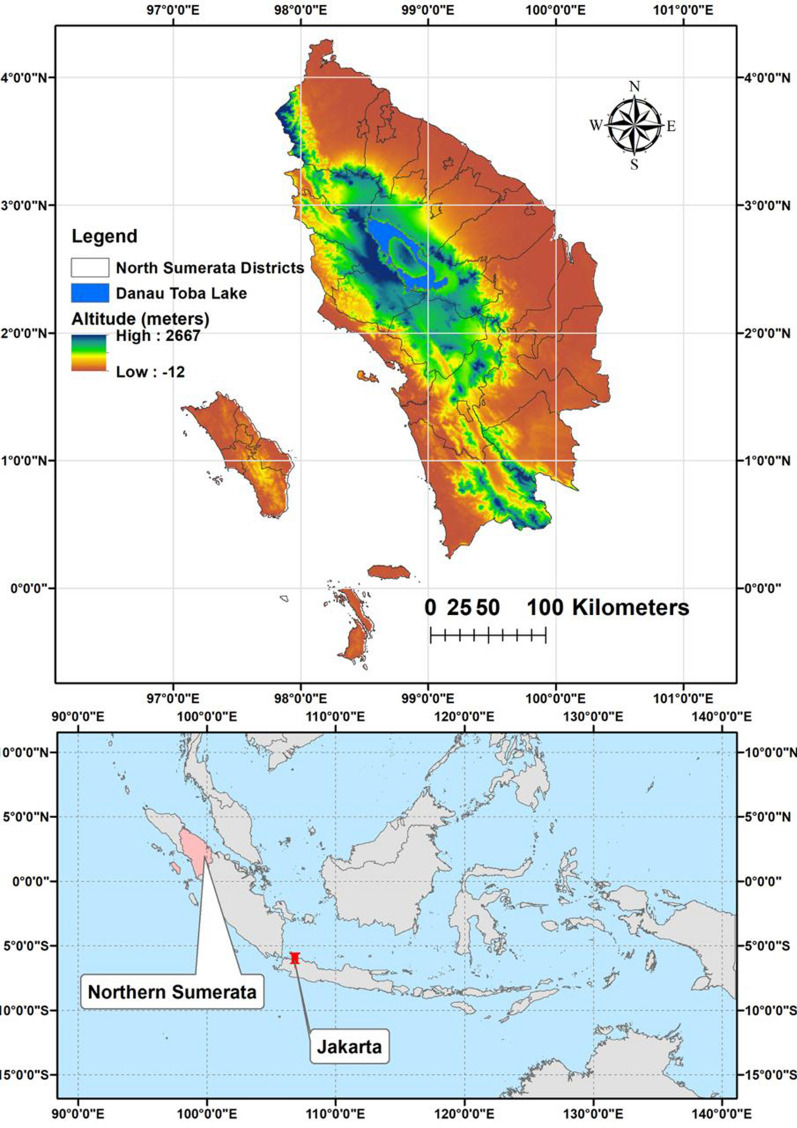
Hot spots (Getis-Ord Gi*) of imported cases in Northern Sumatera, Indonesia from 2019–2020. 2—Batu Bara, 11—Pematangsiantar, 14—Tebing Tinggi, 28—Serdang Bedagai, 29—Simalungun

Twenty-three regencies and cities did not report any indigenous malaria during the study period. The indigenous cases were reported by 10 regencies and the highest cases were reported by Batu Bara (Fig. 3). Imported cases were reported by 14 regencies and the highest case was reported by Asahan and Deli Serdang regencies, respectively (Fig. 4). Indigenous hotspot was reported by Kota Tanjung Balai city and Asahan Regency, respectively (Fig. 5). Imported malaria hotspots were reported in Batu Bara, Kota Tebing Tinggi, Serdang Bedagai and Simalungun (Fig. 6).

### Risk factors of imported malaria

In multivariable logistic regression, ages 19–30, 31–45 and > 45 were more likely to be an imported case compared to the < 13 year age group: 3.3 (adjusted odds ratio [AOR] = 3.31; 95% confidence interval [CI] 1.67, 2.56); 5.7 times (AOR = 5.69; 95% CI 2.65, 12.20), and 5 times (AOR = 5.11; 95% CI 2.41, 10.84). Military personnel, and forest workers and miners were 1154 times (AOR = 197.03; 95% CI 145.93, 9131.56) and 44 times (AOR = 44.16; 95% CI 4.08, 477,93) more likely to be as compared to those working as employees and traders. However, fishermen were 82% less likely to be an imported case (AOR = 0.18, 95% CI 0.08, 0.38) compared to working employees and traders. *Plasmodium falciparum* had lower odds of being imported cases compared to *P. vivax* (AOR = 0.58, 95% CI 0.38, 0.88) (Tables 1,2).

**Table 1:** Socio-demographic characteristics of total and imported cases in North Sumatera, Indonesia, 2019–2020

Characteristic	Total case (2208)	Imported (392)	*P* value
	Number (%)	Number (%)	
Sex			
Female	836 (37.9)	68 (17.4)	< 0.001
Male	1371 (62.1)	324 (82.6)	
Age groups (years)			
≤ 12	475 (21.5)	19 (4.9)	< 0.001
13–18	490 (22.2)	27 (6.9)	
19–30	574 (26.0)	180 (45.9)	
31–45	428 (19.4)	124 (31.6)	
> 45	241 (10.9)	42 (10.7)	
Occupation			
ET	213 (9.7)	51 (13.0)	< 0.001
FG	168 (7.6)	22 (5.6)	
Fisherman	194 (8.8)	9 (2.3)	
FM	10 (0.5)	9 (2.3)	
Housewife	348 (15.8)	42 (10.7)	
Military	193 (8.7)	191 (48.7)	
Students	825 (37.4)	44 (11.2)	
Unemployed	257 (11.6)	24 (6.1)	
Species			
PF	316 (14.3)	115 (29.4)	< 0.001
PV	1613 (73.0)	249 (63.5)	
Mixed	277 (12.6)	28 (7.1)	
Others	2 (0.1)	0 (0.0)	
Diagnosis			
Microscope	1208 (54.7)	296 (75.5)	< 0.001
RDT	1000 (45.3)	96 (24.5)	
Year			
2019	1198 (54.3)	241 (61.5)	< 0.001
2020	1010 (45.7)	151 (9.5)	

ET: employees and traders; FG: farmers and gardeners; FM: forest goers and miners; PF: *P. falciparum,* PV: *P.vivax*; Mixed- Mixed infection of *P. falciparum* and *P. vivax*; RDT- rapid diagnostic test; Others: one *Plasmodium ovale and Plasmodium malariae* each

**Table 2:** Multivariable logistic regression of imported malaria in Northern Sumatera, 2019–2020

Characteristics	Univariate		Multivariable	
	OR	*p* value	AOR	*p* value
Sex				
Female	Ref		Ref	
Male	3.54 (2.68, 4.68)	< 0.001	1.46 (0.93, 2.27)	0.098
Age groups (years)				
< 13	Ref		Ref	
13–18	1.31 (0.72, 2.39)	0.38	1.39 (0.75, 2.58)	0.303
19–30	10.85 (6.62, 17.77)	< 0.001	3.31 (1.67, 6.56)	0.001
31–45	9.72 (5.86, 16.13)	< 0.001	5.69 (2.65, 12.20)	< 0.001
> 45	4.86 (2.75, 8.58)	< 0.001	5.11 (2.41, 10.84)	< 0.001
Occupation				
ET	Ref		Ref	
FG	0.49 (0.28, 0.86)	0.012	0.44 (0.25, 0.78)	0.005
Fisherman	0.16 (0.08, 0.33)	< 0.001	0.18 (0.08, 0.38)	< 0.001
FM	26.82 (3.32, 216.91)	0.002	44.16 (4.08, 477.68)	0.002
Housewife	0.44 (0.28, 0.69)	< 0.001	0.59 (0.33, 1.07)	0.082
Military	569.25 (77.77, 4,166.55)	< 0.001	1,154.35 (145.93, 9,131.56)	< 0.001
Students	0.18 (0.11, 0.28)	< 0.001	0.64 (0.33, 1.27)	0.205
Unemployed	0.35 (0.21, 0.60)	< 0.001	0.96 (0.52, 1.79)	0.91
Species				
PF	Ref		Ref	
PV	0.33 (0.25, 0.43)	< 0.001	0.58 (0.38, 0.88)	0.01
Mixed	0.20 (0.13, 0.31)	< 0.001	0.10 (0.04, 0.27)	< 0.001
Year				
2019	Ref		Ref	
2020	0.62 (0.49, 0.77)	< 0.001	1.68 (1.22, 2.32)	0.001

OR: odds ratio; AOR: adjusted odds ratio; Ref- reference group; ET: employees and traders; FG: farmers and gardeners; FM: forest workers and miners; PF: *P. falciparum,* PV: *P. vivax*, *p*-value significant at < 0.05

### Climatic risk factors of indigenous malaria

Relative humidity and maximum temperature were significant climatic variables for *P. falciparum.* While the minimum temperature was the only significant climatic variable for *P. vivax. Plasmodium falciparum* increased by 12.1% (95% CrI 5.1%, 20.1%) for 1% increase in relative humidity. One-degree increase of maximum temperature increased *P. falciparum* cases by 21.0% (95% CrI 9.0%, 36.2%). *Plasmodium vivax* decreased by 0.8% (95% CrI 0.2%, 1.3%) and 16.7% (95% CrI 13.3%, 19.9%) for each metre and 1 °C increase of altitude and minimum temperature, respectively (Table 3).

**Table 3:** Regression coefficients and 95% CrI from Bayesian models of *Plasmodium falciparum* and *Plasmodium vivax* cases reported by month and regency, North Sumatera, Indonesia, 2019–2020

Covariates	*Plasmodium falciparum*		*Plasmodium vivax*	
	Coefficient (95% CrI)	RR (95% CrI)	Coefficient (95% CrI)	RR (95% CrI)
Intercept*	− 5.79 (− 7.82, − 3.70)		− 6.38 (− 8.14, − 4.45)	
Sex (ref: female)	− 0.011 (− 0.204, 0.180)	0.989 (0.816, 1.197)	− 0.004 (− 0.109, 0.100)	0.996 (0.897, 1.105)
Altitude (meters)	− 0.005 (− 0.012, 0.000)	0.995 (0.988, 1.000)	− 0.008 (− 0.013, − 0.002)	0.992 (0.987, 0.998)^†^
Relative humidity (%)	0.114 (0.050, 0.183)	1.121 (1.051, 1.201)^†^	0.017 (− 0.005, 0.039)	1.017 (0.995, 1.040)
Rainfall (cm)	− 0.002 (− 0.015, 0.011)	0.998 (0.985, 1.011)	0.002 (− 0.005, 0.008)	1.002 (0.995, 1.008)
Maximum temperature (℃)	0.190 (0.086, 0.309)	1.210 (1.090, 1.362)^†^	0.010 (− 0.024, 0.042)	1.010 (0.977, 1.043)
Minimum temperature (℃)	− 0.022 (− 0.102, 0.060)	0.978 (0.903, 1.062)	− 0.182 (− 0.222, − 0.143)	0.833 (0.801, 0.867)^†^
Unstructured random effect	0.0617 (0.0211, 0.1353)		0.05 (0.0187, 0.1037)	

^*^Co-efficient; CrI: credible interval; DIC: deviation information criteria; RR: relative risk

^†^Significant at *p* < 0.05

## Discussion

Nearly one-fifth of malaria cases in Northern Sumatera in 2019 and 2020 were imported malaria. Both indigenous and imported malaria were limited to a few regencies and cities. Risk factors of imported malaria were: > 18 years, military personnel, and forest workers and miners. While fisherman was less likely to be imported malaria compared to employees and traders. Relative humidity and maximum temperature were associated with an increase of *P. falciparum* while altitude and minimum temperature were associated with a decrease in *P. vivax*.

Importation of malaria continues to be an important impediment of malaria elimination efforts [12, 24, 25], driven by complex and multi-faceted factors [25]. Economic migration for better economic, work and social opportunities are important drivers of imported malaria [17, 26–30]. As the country progresses towards malaria elimination, imported malaria becomes the main malaria type [31]. Imported malaria can reintroduce in areas/regions where malaria has been eliminated [32]. Therefore, to prevent the contribution of imported malaria on local transmission in malaria receptivity areas, malaria surveillance including access to prompt diagnosis and treatment to the returning travellers along the international borders should be strengthened [33, 34].

The hot spots of indigenous and imported malaria were located in the east part of Northern Sumatera. The Eastern part of North Sumatera is a coastal area with dense population compared to other parts of North Sumatera. The areas are surrounded by swamps and rivers, and the Hindia ocean. In addition, the main occupation of people in this region is fishermen and farmers [35]. These groups are at a higher risk for malaria infection [36]. Therefore, high coverage of long-lasting insecticidal nets (LLINs) along with prompt diagnosis and treatment need to be strengthened for to curb indigenous malaria in these hotspot regencies.

Military personnel, forest workers and miners were risk factors for imported malaria [37, 38]. As reported earlier military personnel deployed in Papua were responsible for imported malaria in this study [38]. Therefore, strengthening service points for screening and surveillance in collaboration with relevant organizations like the department of military should be undertaken [39, 40]. This includes provision of LLIN to the military personnel deployed at high malaria risk areas, health education, prompt diagnosis and treatment, and screening for malaria after deployment. Malaria prophylaxis to military can be used as an adjunct in addition to other preventative measures to reduce malaria infection in military personnel [40].

Similarly, forest goers and miners continue to be important risk factors of imported malaria in malaria eliminating countries [39]. Therefore, targeting these high-risk groups will be imperative in pursue of malaria elimination [38, 40]. Further, screening posts can be set up at border crossings and migration portals such as taxi stands, and public bus and boat terminals for forest goers and miners [12, 41]. Intervention packages including LLINs, long-lasting insecticidal hammocks (LLIHs), pamphlets on malaria (signs and symptoms) and possible contact points for malaria diagnosis and treatment services in the destination area and education on malaria prevention can be disseminated through these portals. Further, free screening and treatment for asymptomatic malaria can be offered for both returning and travelling migrants [42]. Therefore, malaria control programmes need to integrate these novel methods to target the imported malaria cases. Other novel approaches such as the Malakit used for malaria self-care by the gold miners in French Guiana can be tested for Indonesia [43]. The Malakit supports illegal gold miners to self-diagnosis malaria and supports treatment for *P. falciparum*.

*Plasmodium falciparum* transmission was associated with maximum temperature. Similar findings have been reported in other studies [23]. Temperature plays a crucial role in the transmission cycle of the malaria parasite and mosquito survival [44, 45]. Studies found that at a temperature of 22 °C, the life cycle of malaria parasite development in mosquito vectors is completed in less than 3 weeks [46]. The biting rate and gonotrophic processes are also temperature dependent [47, 48]. Other studies have reported rainfall as an important driver of malaria transmission [49, 50]. However, rainfall was not associated with malaria transmission in this study. *Plasmodium vivax* was inversely associated with altitude and minimum temperature, which is in concordance with other published papers [51–54]. This can partly be due to the detrimental effects of temperature < 16 °C for mosquito survival and *P. vivax*. The temperature also decreases with the increasing altitude.

The limitations of this study are: the completeness and representativeness of surveillance data could not be ascertained. Secondly, sub-microscopic or asymptomatic malaria which could not be captured by surveillance can continue to transmit transmission [55]. Thirdly, unmeasured risk modifiers, such as socio-economic development, living standards, treatment, localised behavioural patterns, population mobility, bed net use and residual indoor insecticide coverage were unaccounted for in this study.

## Conclusion

One-fifth of malaria in Northern Sumatera were imported malaria. Both indigenous and imported malaria hot spots were in the east part of Northern Sumatera. Strengthening surveillance of indigenous malaria, prompt diagnosis and treatment, and provision of good coverage of LLINs in needed in pursue of malaria elimination efforts. Imported malaria can be combatted by expanding control measures such as malaria prophylaxis and other preventive measures through co-ordination with the relevant organizations.

## Supplementary Information

**Additional file 1.** Appendix Table 1. Model selection for *Plasmodium falciparum.* Appendix Table 2. Model selection for *Plasmodium vivax*

## Data Availability

The datasets generated during and/or analysed for this current study will be made available from the corresponding author on reasonable request.

## Abbreviations

AIM: *Action and Investment to Defeat Malaria 2016–2030*
AOR: Adjusted odds ratio
CI: Confidence interval
GIS: Geographical Information Systems
GTS: *Global Technical Strategy for Malaria 2016–2030*
LLIHs: Long-lasting insecticidal hammocks
LLINs: Long-lasting insecticidal nets
RR: Relative risk
SEAR: South-East Asia Region
SMRs: Standardized morbidity ratios
WHO: World Health Organization
ZIP: Zero-inflated Poisson
