# Correction: Spatial analysis to evaluate risk of malaria in Northern Sumatera, Indonesia

**DOI:** 10.1186/s12936-022-04285-5

**Published:** 2022-11-15

**Authors:** Fahmi Fahmi, Ayodhia Pitaloka Pasaribu, Minerva Theodora, Kinley Wangdi

**Affiliations:** 1 Department of Electrical Engineering, Universitas Sumatera Utara, Medan, 20155 Indonesia; 2 Department of Child Health, Medical Faculty, Universitas Sumatera Utara, Medan, 20155 Indonesia; 3 Directorate of Vector Borne and Zoonotic Disease Control, Ministry of Health of Indonesia, South Jakarta, 12950 Indonesia; 4 Department of Global Health, National Centre for Epidemiology and Population Health, College of Health and Medicine, Australian National University, Acton, ACT 2601 Australia

## Correction to: Malaria Journal (2022) 21:241 https://doi.org/10.1186/s12936-022-04262-y

Following publication of the article [1], the authors flagged that out-of-date figures had been provided. The article has since been corrected with up-to-date, correct versions of the figures, and these correct figures are provided in this erratum (Figs. 1, 2, 3, 4, 5 and 6).

**Fig. 1:**
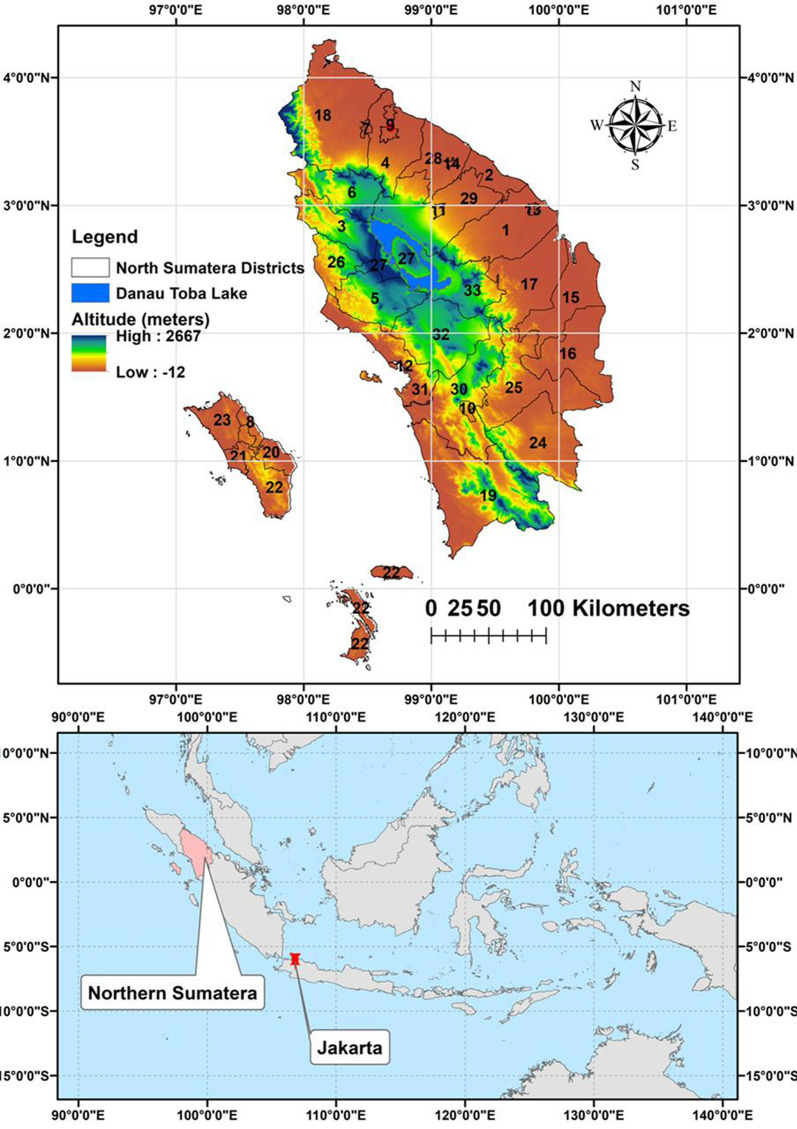
Map of Northern Sumatera, Indonesia with malaria transmitting regencies. 1—Asahan, 2—Batu Bara, 3—Dairi, 4—Deli Serdang, 5—Humbang Hasundutan, 6—Karo, 7—Binjai, 8—Gunungsitoli, 9—Medan, 10—Padangsidimpuan, 11—Pematangsiantar, 12—Sibolga, 13—Tanjungbalai, 14—Tebing Tinggi, 15—Labuhanbatu, 16—Labuhanbatu Selatan, 17—Labuhanbatu Utara, 18—Langkat, 19—Mandailing Natal, 20—Nias, 21—Nias Barat, 22—Nias Selatan, 23—Nias Utara, 24—Padang Lawas, 25—Padang Lawas Utara, 26—Pakpak Bharat, 27—Samosir, 28—Serdang Bedagai, 29—Simalungun, 30—Tapanuli Selatan, 31—Tapanuli Tengah, 32—Tapanuli Utara, 33—Toba

**Fig. 2:**
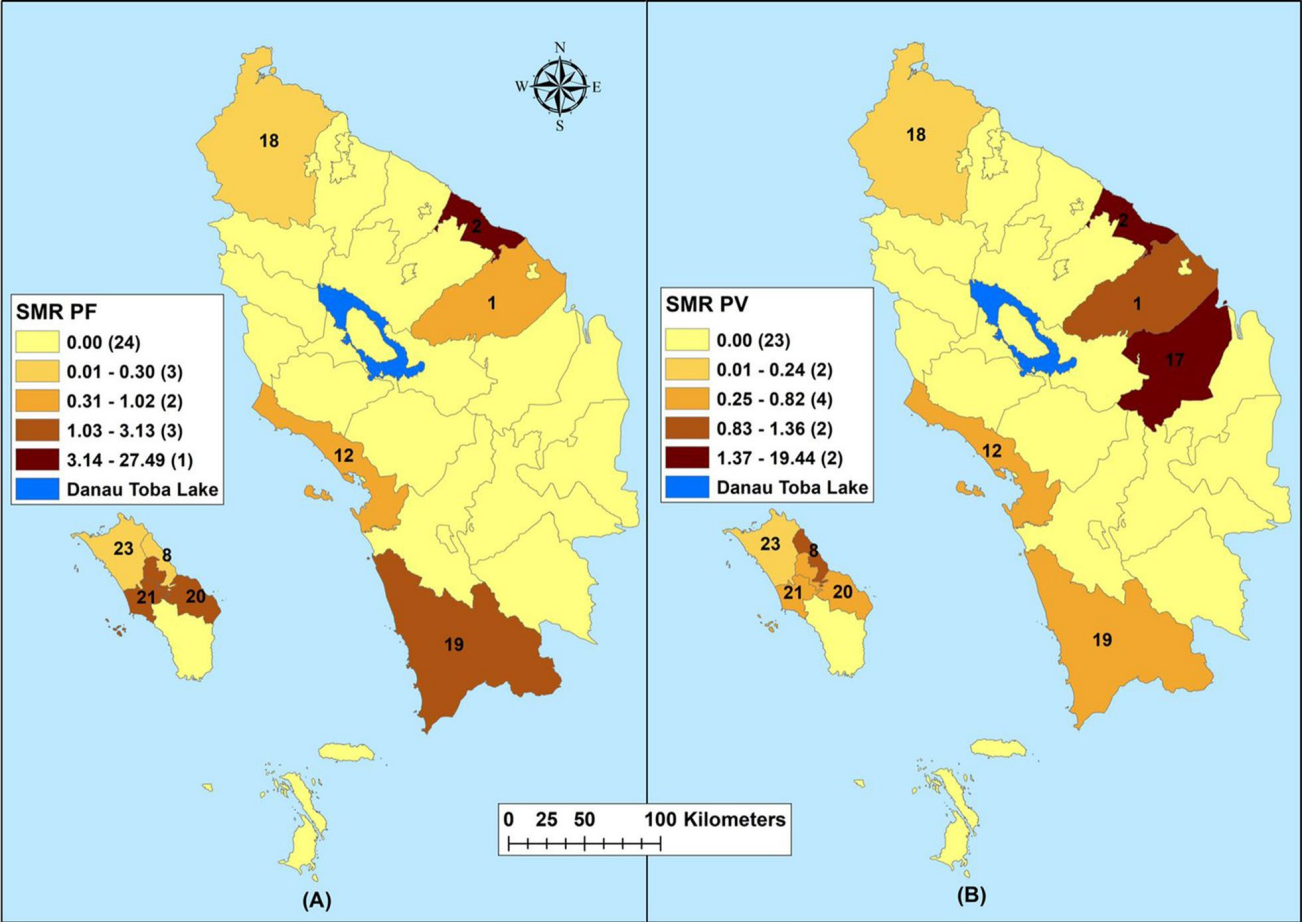
Raw standardized morbidity ratios of **A** *Plasmodium falciparum* and **B** *Plasmodium vivax* by regencies in Northern Sumatera, Indonesia from 2019–2020. 1—Asahan, 2—Batu Bara, 8—Gunungsitoli, 12—Sibolga, 17—Labuhanbatu, 18—Langkat, 19—Mandailing Natal, 20—Nias, 21—Nias Barat, 23—Nias Utara

**Fig. 3:**
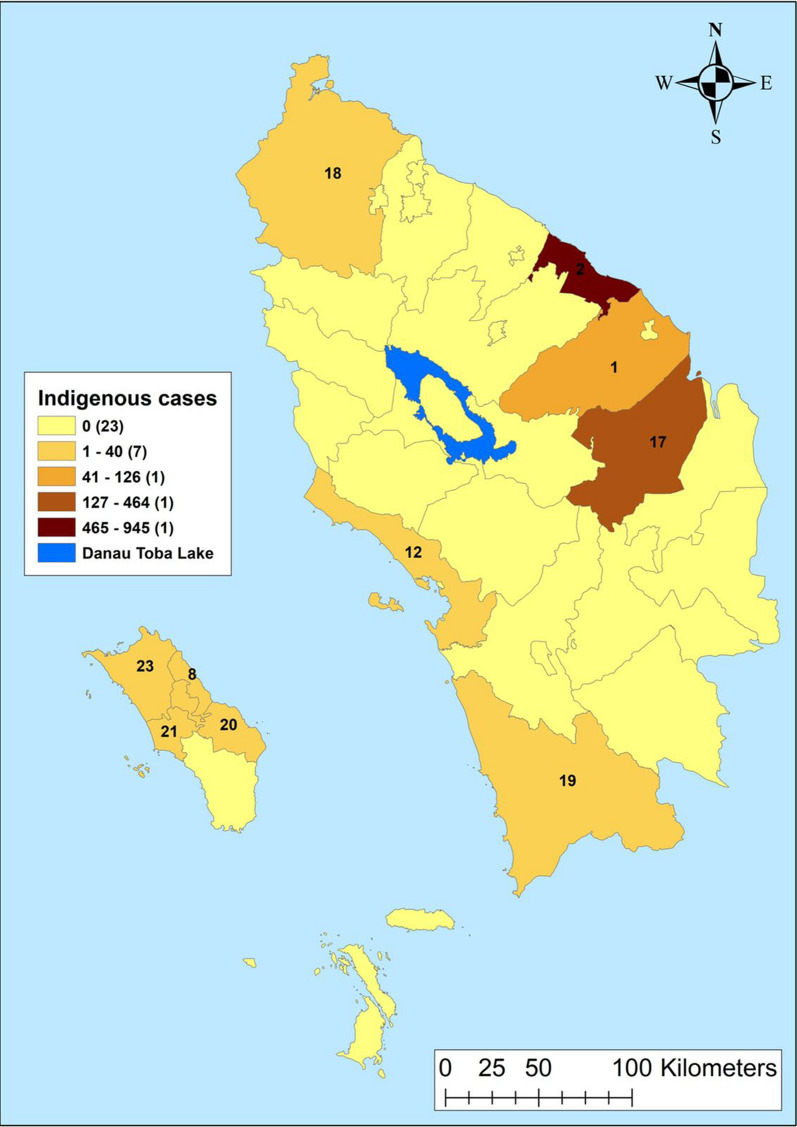
Indigenous cases by regencies in Northern Sumatera, Indonesia from 2019–2020. 1—Asahan, 2—Batu Bara, 8—Gunungsitoli, 12—Sibolga, 17—Labuhanbatu, 18—Langkat, 19—Mandailing Natal, 20—Nias, 21—Nias Barat, 23—Nias Utara

**Fig. 4:**
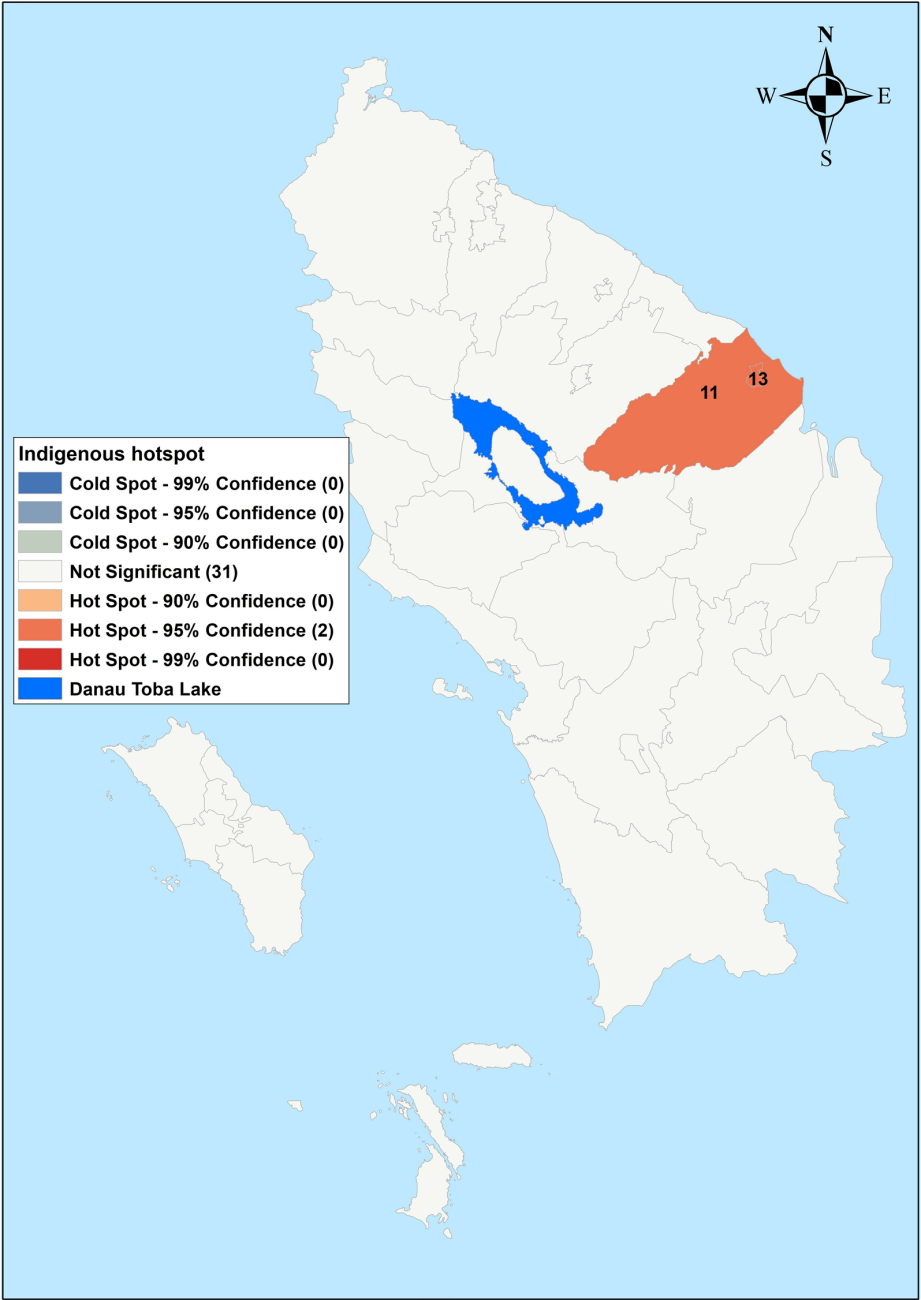
Hot spots (Getis-Ord Gi*) of indigenous cases in Northern Sumatera, Indonesia from 2019–2020. 11—Pematangsiantar, 13—Tanjungbalai

**Fig. 5:**
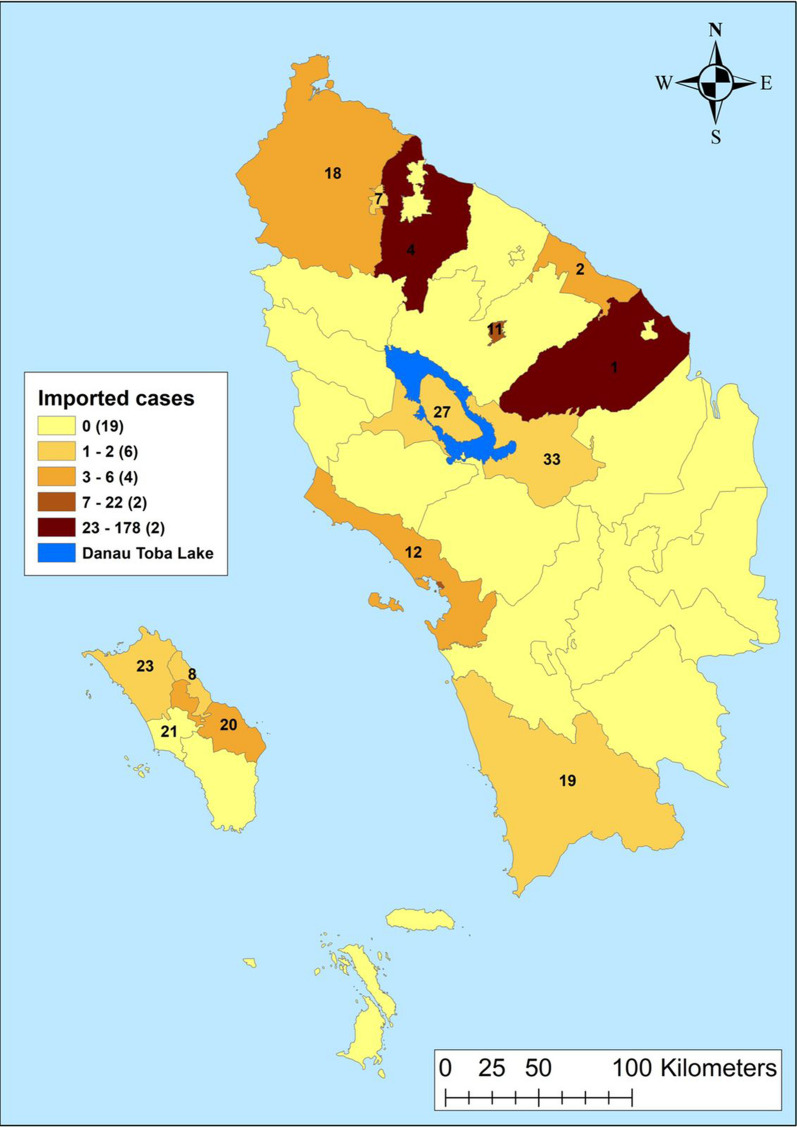
Imported cases by regencies in Northern Sumatera, Indonesia from 2019–2020. 1—Asahan, 2—Batu Bara, 4—Deli Serdang, 7—Binjai, 8—Gunungsitoli, 11—Pematangsiantar, 12—Sibolga, 18—Langkat, 19—Mandailing Natal, 20—Nias, 21—Nias Barat, 23—Nias Utara, 27—Samosir, 33—Toba

**Fig. 6:**
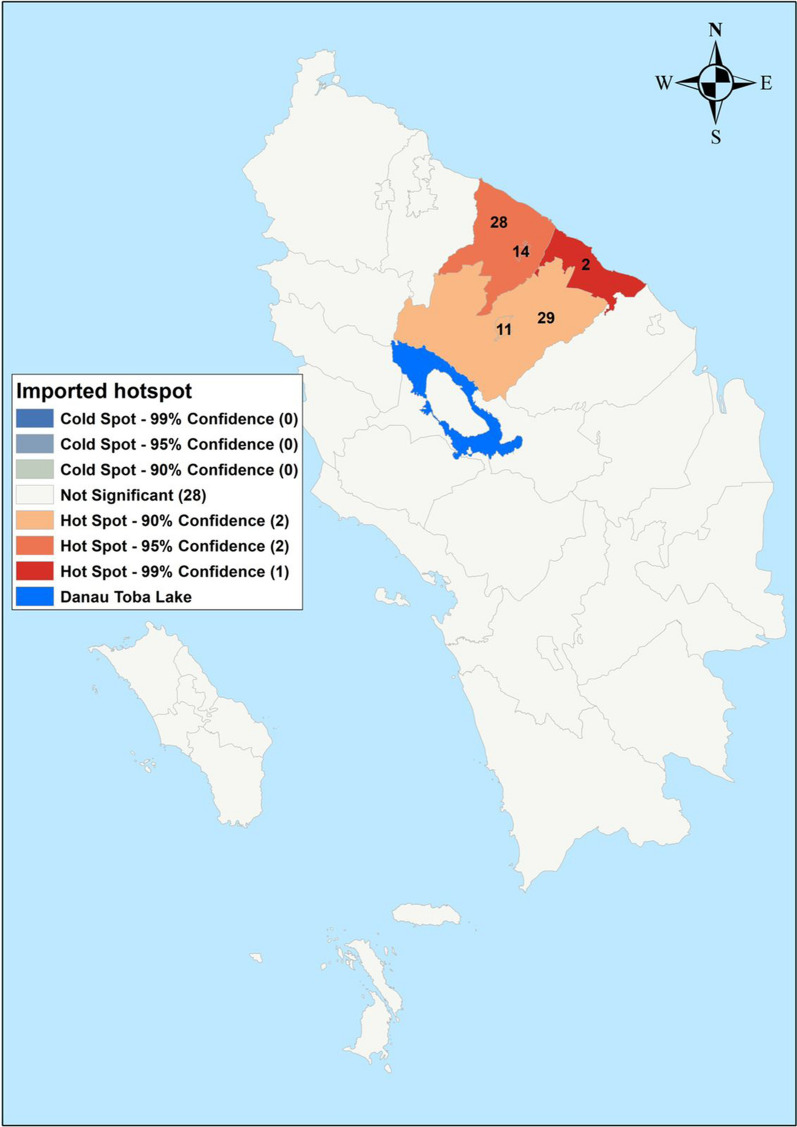
Hot spots (Getis-Ord Gi*) of imported cases in Northern Sumatera, Indonesia from 2019–2020. 2—Batu Bara, 11—Pematangsiantar, 14—Tebing Tinggi, 28—Serdang Bedagai, 29—Simalungun

The authors thank you for reading and apologize for any inconvenience caused.
