# Supplementary material for: Spatial analysis to evaluate risk of malaria in Northern Sumatera, Indonesia
Source: Malar J. 2022 Aug 20;21:241. doi: 10.1186/s12936-022-04262-y (PMC9392258; doi:10.1186/s12936-022-04262-y)
Supplement: Supplementary file 1 — Additional file 1. Appendix Table 1. Model selection for Plasmodium falciparum. Appendix Table 2. Model selection for Plasmodium vivax [file 12936_2022_4262_MOESM1_ESM.pdf]

Appendix

Appendix Table 1 Model selection for *Plasmodium falciparum*

Model	N	AIC	BIC
Poisson	789	7841.507	7864.861
ZIP	792	1108.984	1141.706

Appendix Table 2 Model selection for *Plasmodium vivax*

Model	N	AIC	BIC
Poisson	789	7841.507	7864.861
ZIP	792	1869.382	1902.104
